# A Bizarre, Unexplained, and Progressive External Rotation of the Shoulder as a Presentation of a Metastatic Deposit in the Rotator Cuff

**DOI:** 10.1155/2015/962931

**Published:** 2015-10-12

**Authors:** Sherif El-Tawil, Aditya Prinja, Jeremy Stanton

**Affiliations:** ^1^Department of Orthopaedics, Princess Alexandra Hospital, Hamstel Road, Harlow, Essex CM20 1QX, UK; ^2^Department of Orthopaedics, Colchester Hospital, Turner Road, Colchester CO4 5JL, UK

## Abstract

We describe the first reported case of a tumour deposit within the rotator cuff presenting as a bizarre, progressive, and fixed external rotation deformity of the shoulder. It is also the first reported case to our knowledge of an oesophageal primary metastasising to the rotator cuff.

## 1. Introduction

Malignant tumour metastasis to skeletal muscle is extremely rare, but in these rare cases, spinal and shoulder musculature tend to be affected [1]. They are usually detected as incidental findings on staging computerised tomography (CT). This case report describes a progressive and painful deformity of the shoulder due to tumour metastasis to the infraspinatus muscle from an oesophageal adenocarcinoma. The resulting bizarre deformity has never been described in the literature and was a diagnostic puzzle for both the surgeon and oncologist. This is also, to the best of our knowledge, the first ever description of an oesophageal primary metastasising to the rotator cuff.

## 2. Case Presentation

**Figure 1:**
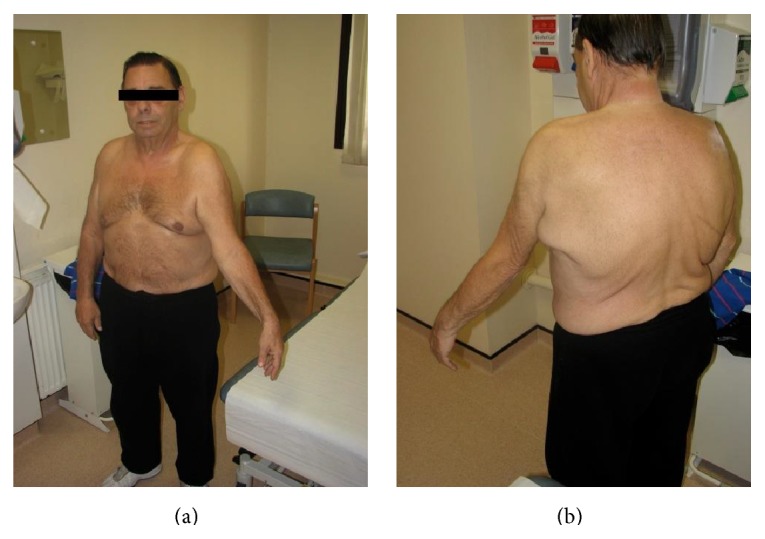
Patient on presentation, with fixed external rotation deformity of the left upper limb.

A 63-year-old right hand dominant gentleman presented to our orthopaedic clinic with a six-month history of an unusual, progressive, painful, and fixed external rotation deformity of his left shoulder (Figures 1(a) and 1(b)).

One year previously, he was diagnosed with oesophageal adenocarcinoma, which was treated with a course of neoadjuvant chemotherapy followed by oesphagectomy. Preoperative staging CT had shown no evidence of distant metastases. Histology revealed a poorly differentiated adenocarcinoma with focal perineural invasion but clear resection margins. He had declined adjuvant chemoradiotherapy.

The patient initially experienced reduced range of movement in his left shoulder six months following his oesphagectomy and began to notice that self-hygiene was becoming troublesome. He was referred to the physiotherapists who noted a progressive permanent external rotation deformity of his left shoulder with a fixed abduction of 30 degrees and a maximum abduction of 90 degrees. He complained of a continuous pain from the shoulder radiating to his elbow, and was slightly tender over the infraspinatus muscle bulk, although no mass was palpable. The awkward position of his arm increasingly interfered with washing and dressing. The external rotation deformity worsened and became fixed at 40 degrees, at which point he was referred to the orthopaedic clinic where he presented a great diagnostic challenge.

**Figure 2:**
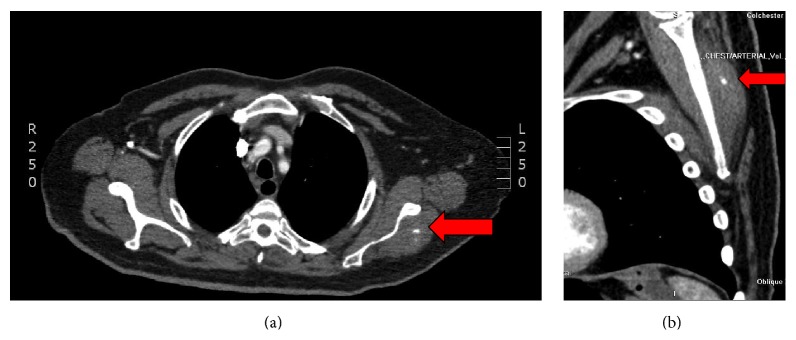
Axial and oblique CT scan, respectively, demonstrating lesion within the left infraspinatus muscle (arrow). Area of central calcification evident.

As the shoulder pain and deformity progressed, CT and MRI were performed to aid the diagnosis. These showed a discrete mass measuring 4 × 4 × 4 cm in the infraspinatus muscle (Figures 2(a) and 2(b)).

Biopsy subsequently confirmed metastatic oesophageal adenocarcinoma with the histology showing infiltration of the infraspinatus muscle by poorly differentiated adenocarcinomatous cells with calcific deposits. Further imaging revealed local recurrence of the primary. The decision was made in liaison with the patient and oncologist to have palliative radiotherapy to his shoulder to help with pain control. However, his shoulder pain deteriorated despite oral morphine and amitriptyline and necessitated a suprascapular nerve block to aid symptom control. The shoulder deformity did not improve, and the patient passed away after 10 months.

## 3. Discussion

Haematogenous spread of malignant tumours to skeletal muscle is extremely rare. Those patients who do have muscular deposits tend to have advanced disease with poorly differentiated primaries [1], as was the case in our patient.

There have been reports of renal cell, lung, and thyroid carcinomas metastasising to the infraspinatus [2–4]. However, this is the first report of an oesophageal primary metastasising to the rotator cuff. Also, to our knowledge no reports have previously described a resulting external rotation deformity such as this.

This case draws attention to the fact that metastases in the rotator cuff can give rise to unusual upper limb positional deformities; recognising this is the first step in achieving a diagnosis in such cases which are rare and previously unreported. This case also highlights the importance of the orthopaedic surgeon and oncologist liaising closely in such rare cases, keeping an open mind on the possible reasons for such a progressive shoulder deformity. The diagnostic puzzle can rapidly be solved by combining a detailed history and examination with imaging in the form of CT or MRI, as long as one recognises that tumour deposits can arise in the rotator cuff in the first instance.

It is also important to recognise the appearance of such lesions on CT as such lesions tend to be incidentally found on initial staging CTs for the primary tumour. The classic appearance is that of a rim-enhancing intramuscular mass with central hypoattenuation or calcification. MRI classically provides superior soft tissue contrast to CT and such muscular tumour deposits appear as a ring of peripheral oedema around the mass [5, 6]. Interestingly, however, the MRI in our patient did not demonstrate the lesion as clearly as CT; the images were degraded possibly because the patient found it difficult to tolerate the externally rotated position of the arm within the tighter scanner.

The infraspinatus muscle is the main external rotator of the shoulder and we hypothesise that the increased bulk created by the metastasis caused the external rotational deformity seen with our patient; the infraspinatus muscle is encased in a thick fibrous fascia which would be resistant to stretch, thereby allowing any tumour to exert direct pressure effects on the muscle itself which is incarcerated within the infraspinatus fossa. Another hypothesis is that it is usual for surrounding stromal tissue to undergo myofibroblastic conversion in the presence of neighbouring tumour and this gives rise to stiff fibrotic tissue as seen in wound healing [7]. This contracted and stiffened tissue could potentially give a shortened and fixed infraspinatus that could contribute to the deformity seen here. Unfortunately we were unable to obtain the original histology slides from the infraspinatus biopsy, to confirm the presence of such myofibroblasts.

The management of such lesions is difficult in view of their location, but as the primary histology is likely to be poorly differentiated with a poor prognosis, as in our patient, the treatment is likely to be palliative. However, further reporting of similar cases would be useful to ascertain other management strategies which obviously are customised towards the specific patient's level of disease.

## References

[1] Pretorius E. S., Fishman E. K. (2000). Helical CT of skeletal muscle metastases from primary carcinomas. *American Journal of Roentgenology*.

[2] Hyodo T., Sugawara Y., Tsuda T. (2009). Widespread metastases from sarcomatoid renal cell carcinoma detected by ^18^F-FDG positron emission tomography/computed tomography. *Japanese Journal of Radiology*.

[3] Maréchal, Rivat P., Leclercq R. (2001). An unusual shoulder stiffness: metastasis in the infraspinatus muscle as the first clinical manifestation of lung carcinoma. *Revue de Chirurgie Orthopedique et Reparatrice de l'Appareil Moteur*.

[4] Fernàndez-Real J. M., Villabona C., Fernândez-Castañer M., Sagarra E., Gómez-Saéz J. M., Soler J. (1996). Expression of ICAM-1 in distant metastatic thyroid carcinoma. *Journal of Endocrinological Investigation*.

[5] Hanna S. L., Fletcher B. D., Parham D. M., Bugg M. F. (1991). Muscle edema in musculoskeletal tumors: MR imaging characteristics and clinical significance. *Journal of Magnetic Resonance Imaging*.

[6] Suto Y., Yamaguchi Y., Sugihara S. (1997). Skeletal muscle metastasis from lung carcinoma: MR findings. *Journal of Computer Assisted Tomography*.

[7] Otranto M., Sarrazy V., Bonté F., Hinz B., Gabbiani G., Desmoulière A. (2012). The role of the myofibroblast in tumor stroma remodeling. *Cell Adhesion and Migration*.

